# NS2A V89F mutation in a DENV1 clinical isolate enhances neurotropism and neuroinvasion

**DOI:** 10.3389/fcimb.2026.1922765

**Published:** 2026-08-26

**Authors:** Yuqi Zhao, Linqing Wang, Yifan Lin, Yangyang Chen, Yan Zhan, Kexin Xi, Yingfang Liu, Chenguang Shen, Bao Zhang, Weiwei Xiao, Qinghua Wu, Li Zhu, Wei Zhao, Fan Yang, Weijin Gong, Yuntao Lu, Jianhai Yu

**Affiliations:** 1 Department of Neurosurgery, Nanfang Hospital, Southern Medical University, Guangzhou, Guangdong, China; 2 BSL-3 Laboratory (Guangdong), Guangdong Provincial Key Laboratory of Tropical Disease Research, Key Laboratory of Infectious Diseases Research in South China, School of Public Health, Southern Medical University, Guangzhou, Guangdong, China; 3 Biosafety Level 3 Laboratory, Medical School, Shenzhen University, Shenzhen, Guangdong, China; 4 Shenzhen Center for Disease Control and Prevention, Shenzhen, Guangdong, China; 5 Department of Infectious Diseases, Foshan Fourth People’s Hospital, Foshan, Guangdong, China

**Keywords:** dengue virus, neuroinvasion, neurotropism, NS2A, putative adaptive mutation, structural remodeling

## Abstract

**Introduction:**

Dengue virus (DENV) neurological complications are increasingly reported, yet the viral genetic determinants of neurotropism remain poorly characterized.

**Methods:**

We screened 25 DENV1 clinical isolates from the 2014 outbreak in Guangdong, China, for neurotropism in suckling mice, and integrated comparative genomics, pre-expression functional assays, population-scale sequence analysis, and OpenFold3 structural modeling to identify mutations associated with enhanced neuroinvasion.

**Results:**

We found that only strain P1253 induced neurological symptoms and mortality via subcutaneous inoculation, producing cortical-selective lesions distinct from the diffuse encephalitic damage observed after intracranial inoculation, and P1253 replicated preferentially in human brain microvascular endothelial cells (HBMEC) compared to contemporaneous strains. Comparative genomics identified three unique mutations in P1253 (NS1 175Y→H, NS2A 89V→F, NS4A 2V→I), and pre-expression assays demonstrated that only NS2A 89V→F significantly enhanced viral replication and cytopathic effect in HBMEC. Analysis of 1,990 complete DENV1 genomes revealed five natural mutant types in the NS2A 89 -96 residue region, with P1253 representing the FIPI quadruple-mutant type, and OpenFold3 structural prediction showed that 89V→F introduced on the VIPI background induced the most significant distal domain reorientation (RMSD 1.605 Å), increasing the centroid-to-centroid distance between residues 89 -96 and 185 -218 from 18.221 Å to 27.462 Å.

**Discussion:**

These findings identify NS2A 89V→F as a candidate adaptive mutation associated with enhanced neurotropism in DENV1 and provide a framework for monitoring neurovirulent variants.

## 1 Introduction

Dengue fever is a mosquito-borne infectious disease transmitted to humans through the bite of infected Aedes mosquitoes. As the most widely distributed vector-borne virus globally, dengue poses an escalating transmission risk with climate warming and expanding mosquito ranges (Romanello et al., 2024). Currently, over half the population in more than 100 countries faces infection risk, and global cases exceeded 14 million in 2024 (World Health Organization). The World Health Organization has listed DENV among the pathogens most likely to spark the next pandemic (Mallapaty, 2024).

The updated clinical guidelines classify dengue into three categories: dengue without warning signs (D), dengue with warning signs (DWS), and severe dengue (SD). DENV-associated neuropathology is now recognized as a clinical indicator of SD (Htun et al., 2021), and patients with neurological manifestations carry a higher risk of poor prognosis. Epidemiological surveys across multiple regions have confirmed the contribution of DENV neuroinvasive disease, with acute encephalitis syndrome studies identifying DENV as a significant etiological agent (Ngwe Tun et al., 2020a; Ravi et al., 2022). In recent years, the traditional view that DENV causes only fever and hemorrhage has been significantly revised. Multiple studies have confirmed that DENV can invade the central nervous system (CNS) and cause severe neurological complications such as encephalitis, encephalopathy, acute flaccid paralysis, and Miller Fisher syndrome (de Silva et al., 2019; Prabhat et al., 2020; Bentes et al., 2021); neuroimaging studies show that NeuroDengue patients often exhibit MRI lesions in the thalamus, basal ganglia, and cortex (Lnu et al., 2022); and epidemiological data indicate higher cerebrospinal fluid (CSF) detection rates for DENV-2 and DENV-3, suggesting greater neurotropic potential (Noronha et al., 2025).

The blood-brain barrier (BBB) constitutes an obligatory checkpoint for viruses entering the CNS (Kadry et al., 2020; Rani et al., 2024). DENV can directly infect BBB-associated cells *in vitro*, and virus-induced vascular leakage may further facilitate barrier breach (Calderón-Peláez et al., 2019; Puerta-Guardo et al., 2025). However, the mechanisms underlying DENV neurological disease remain poorly understood. Viral direct infection of the CNS, host immune responses, and infection-induced vascular leakage are thought to act in concert (Bentes et al., 2021; Trivedi and Chakravarty, 2022). Notably, neurological sequelae of dengue are increasingly recognized in large cohort studies (Lin et al., 2024), suggesting that viral factors may influence BBB integrity during severe infection (Araújo et al., 2012), and DENV neuroinvasiveness may increase with viral adaptive evolution.

However, clinical strains with confirmed neurotropism and suitable animal models remain scarce, and differences in neurological damage between strains are incompletely defined (Bastos et al., 2018; Dhenni et al., 2018). In 2014, Guangdong Province experienced the most severe dengue fever outbreak on record, reporting 45224 cases and 6 deaths. Our previous work established that DENV1 was the predominant serotype and formed stable evolutionary lineages (Yu et al., 2019). In the course of preparing brain tissue from 25 DENV1 isolates collected that year, we identified strain P1253 as potentially neurotropic. This strain caused neurological symptoms and lethality in Kunming suckling mice after subcutaneous multi-point injection, suggesting an ability to invade the BBB and induce neurological disease. Preliminary comparative genomics further revealed that P1253 carried unique amino acid substitutions in non-structural proteins (NS1, NS2A, and NS4A) relative to non-neurovirulent contemporaneous strains, raising the hypothesis that mutations in these replication-associated proteins may contribute to its enhanced neuroinvasive phenotype.

Flavivirus non-structural proteins are increasingly recognized as critical modulators of viral replication efficiency and host-cell tropism. Secreted NS1 has been shown to trigger endothelial dysfunction and increase vascular permeability, potentially facilitating virus dissemination across the BBB (Puerta-Guardo et al., 2025). NS2A is a transmembrane protein that spans the endoplasmic reticulum membrane and functions as a scaffold for the viral replication complex; its transmembrane segments have been implicated in modulating viral RNA synthesis and cytopathic effects in a cell-type-dependent manner (Xie et al., 2013; Wu et al., 2017). NS4A induces membrane remodeling to form replication organelles and contributes to the assembly of functional replication complexes. Consequently, amino acid substitutions in these non-structural proteins represent plausible candidate determinants of cell-type-specific replication efficiency. In this study, we therefore included NS1, NS2A, and NS4A in our comparative genomics screen to identify mutations associated with the observed neuroinvasive phenotype, followed by functional validation of each candidate.

Here, we further characterize the neurotropism of P1253 using Kunming and C57BL/6 suckling mouse models, analyze its infection mechanism in HBMEC as a BBB component, and integrate comparative genomics with protein structural modeling to identify mutations associated with enhanced neuroinvasion and to explore their structural features and distribution in circulating DENV strains.

## 2 Materials and methods

### 2.1 Viruses and cells

C6/36, Vero, HBMEC, and BHK-21 cells were maintained in our laboratory. The 25 DENV1 clinical isolates used in this study were obtained from the virus strain repository of the Guangdong Provincial Center for Disease Control and Prevention (GDCDC), which had originally isolated these strains from patient sera during routine dengue surveillance and outbreak response in 2014. Our study used these pre-isolated viral stocks for animal and cell culture experiments only and did not involve the direct collection or analysis of human tissues, sera, or identifiable patient data. The clinical background of the corresponding patients and the full-genome sequences have been reported previously (Yu et al., 2019) and deposited in GenBank. The detailed patient information and accession numbers are provided in **Supplementary Table 1**. Strain P1253 was isolated from a 25-year-old female patient with mild dengue fever who showed no neurological symptoms or sequelae on follow-up. P1258, P1260, and P1278, which were injected subcutaneously into mice at multiple points without death, were selected as representative non-neurovirulent strains for cell experiments. The DENV1 Hawaii strain was included as a reference standard strain control in cell susceptibility assays for comparison with the 2014 Guangdong clinical isolates. Viral titers were determined by the 50% tissue culture infectious dose (TCID50) assay in BHK-21 cells and calculated using the Reed-Muench method.

### 2.2 Animal experiments

Specific-pathogen-free (SPF) 1-day-old Kunming and C57BL/6 suckling mice were purchased from the Laboratory Animal Center of Southern Medical University. All animal experiments were approved by the Laboratory Animal Ethics Committee of Southern Medical University (approval number: 2012-041) and conducted in strict accordance with institutional guidelines for the care and use of laboratory animals.

Twenty-five DENV1 strains were used to infect Kunming suckling mice via intracranial injection or subcutaneous multi-point injection at a dose of 10^5^ TCID50 per mouse. C57BL/6 suckling mice were infected with P1253 only, via both intracranial and subcutaneous routes, to confirm neurotropism. The control group received an equal volume of sterile PBS.

After infection, mice were monitored for 15 days. Clinical symptoms and signs were recorded, and survival curves were plotted. Experiments were repeated until more than 20 mice per strain per route were infected. Moribund mice and mice that did not develop disease were necropsied to collect brain tissue. Brains from P1253-infected C57BL/6 mice (both routes) were sent to Guangzhou Huayin Medical Science Company Limited (Guangzhou, China) for tissue sectioning and histopathological analysis.

### 2.3 Infection kinetics of DENV1 isolates in cell models

C6/36, Vero, and HBMEC cells were seeded into 6-well plates (Sangon Biotech, Shanghai, China) and infected with DENV1 isolates at a multiplicity of infection (MOI) of 0.5. After a 2-hour incubation, maintenance medium containing 2% fetal bovine serum (FBS) was added. Cell culture supernatants (300 μL per well) were collected daily for 5 consecutive days, and viral loads were measured.

### 2.4 Viral RNA extraction and quantification

Total RNA was extracted from mouse brain homogenates and cell culture supernatants using the QIAamp Viral RNA Mini Kit (Qiagen, Hilden, Germany). cDNA was synthesized with the PrimeScript II First Strand cDNA Synthesis Kit (TaKaRa Bio, Shiga, Japan). RT-qPCR was performed using the Pro Taq HS Premix Probe qPCR Kit (Hunan Accurate Biotechnology Co., Ltd.). Primers and probes were synthesized by Sangon Biotech (Shanghai, China). The sequences were: DENV1 probe: 5′-FAM-ACAGCCAGTGTTCCAGTCATCAGCA-Eclipse-3′;

Forward primer: 5′-GATGAGATCCAGATGGAGTAGAAAG-3′;
Reverse primer: 5′-TCAGTTGTCCCATTATAAGAAGGAG-3′.

### 2.5 Susceptibility monitoring of DENV1 isolates

Real-time cell analysis (RTCA) was performed using the iCELLigence System (ACEA Biosciences, San Diego, CA, USA) in HBMEC as a BBB-related model. HBMEC and C6/36 cells were seeded into 8-well plates and monitored every 10 min for 24 h. The virus (MOI = 1) was then added, and impedance was recorded every 1 min for 2 h. The virus inoculum was replaced with maintenance medium containing 2% FBS, and monitoring continued every 15 min for 168 h. Data were analyzed with RTCA software.

### 2.6 Mutational profiling and construction of protein expression vectors

Full-length genome sequences of five representative DENV1 strains (P1253, P1257, P1258, P1278, and P1346), selected to cover the major geographic locations of the 2014 Guangdong outbreak, were determined by Illumina sequencing of overlapping RT-PCR amplicons and deposited in GenBank in previous report (Yu et al., 2019). The E gene sequences of all 25 clinical isolates used in this study were also sequenced and deposited in GenBank. The sequences were aligned using BioEdit v7.7.1 (https://www.bioedit.com/) to identify mutations. Detailed sequencing status and accession numbers for all 25 strains are provided in **Supplementary Table 1**.

The NS1, NS2A, and NS4A genes of P1253 were synthesized and cloned into the eukaryotic expression vector pEGFP-N1. NS1 and NS4A were inserted between BamHI and XhoI sites; NS2A was inserted between EcoRI and HindIII sites. Plasmids were transformed into Escherichia coli, and positive clones were verified by colony PCR using pEGFP-N1 universal primers (pEGFP-N-3′: CGTCGCCGTCCAGCTCGACCAG; pEGFP-N-5′: TGGGAGGTCTATATAAGCAGAG). For each construct, five independent positive colonies were subsequently verified by Sanger sequencing, respectively. Sequencing covered the full-length insert regions, including the mutation sites, and confirmed 100% identity with the designed reference sequences without any unintended mutations. Verified plasmids were stored at −80 °C. The original sequencing trace files are provided in **Supplementary Data S3**.

### 2.7 Pre-expression assay in HBMEC

To investigate whether pre-expression of P1253-derived NS proteins could modulate P1258 replication in HBMEC, HBMEC cells were seeded into 8-well plates and monitored by RTCA every 10 min for 24 h. pEGFP-N1-NS1, pEGFP-N1-NS2A, pEGFP-N1-NS4A, or empty pEGFP-N1 were transfected into HBMEC using Lipofectamine 3000 (Thermo Fisher Scientific). Impedance was recorded every 10 min during the 8-hour transfection period and every 15 min for 48 h post-transfection. GFP expression was examined at 24 and 48 h; when GFP-positive cells exceeded 50% in all groups, P1258 virus (MOI = 1) was added. Impedance was recorded every 1 min for 2 h, then every 15 min for 168 h after medium replacement. Cell culture supernatants were collected at designated time points for qPCR, and cytopathic effect (CPE) was observed daily.

### 2.8 Sequence diversity analysis and protein structure prediction of DENV1 NS2A

A dataset of DENV1 NS2A sequence diversity was created from the NCBI Virus database (https://www.ncbi.nlm.nih.gov/labs/virus/vssi/#/). As of June 18, 2026, 1,990 complete DENV1 genome sequences were retrieved using the search terms “dengue virus type 1” and “Complete” for nucleotide sequences. Multiple sequence alignment was performed with MAFFT version 7 (https://mafft.cbrc.jp/alignment/server/index.html), and NS2A coding sequences were extracted using BIOEdit v7.7.1. Metadata on geographic origin and collection year were compiled to characterize the distribution of the NS2A V89F mutation.

To assess the structural impact of progressive NS2A 89–96 mutations, full-length sequences of the five natural mutant types (VLRL to FIPI) were submitted to OpenFold3 (Tamarind Bio online platform, https://app.tamarind.bio/openfold) for template-free *de novo* prediction. OpenFold3 generated five models per sequence. The model confidence metrics (avg_pLDDT, GPDE, pTM) for all models are provided in **Supplementary Table 2**, and the top-ranked model (highest avg_pLDDT) for each mutant type was selected for downstream analysis. The specific protein structure file can be found in **Data S1**. Structures were visualized in Discovery Studio v2016 (https://www.3ds.com/products/biovia/discovery-studio), and the Align Structures module was used to compare structures with superimposing stepwise in the mutational accumulation order. The global backbone conformational divergence for each pairwise alignment was quantified by the root-mean-square deviation (RMSD) of Cα atoms. The centroid-to-centroid distances between the 89–96 and 185–218 regions were calculated to evaluate distal domain reorientation.

### 2.9 Statistical analysis

All experiments were repeated independently at least three times. Survival curves were analyzed by the Kaplan-Meier method and compared using the log-rank (Mantel-Cox) test. Viral loads and RTCA cell index values were compared by one-way ANOVA followed by Tukey’s multiple comparisons test. For multi-day infection kinetics, viral loads at each daily time point (Day 0 to Day 5) were independently compared among strains. GraphPad Prism 9.0 was used. P < 0.05 was considered statistically significant (* P < 0.05, ** P < 0.01, *** P < 0.001).

## 3 Results

### 3.1 Clinical isolate DENV1 P1253 crosses the blood-brain barrier and causes neurological symptoms in mice

**Figure 1:**
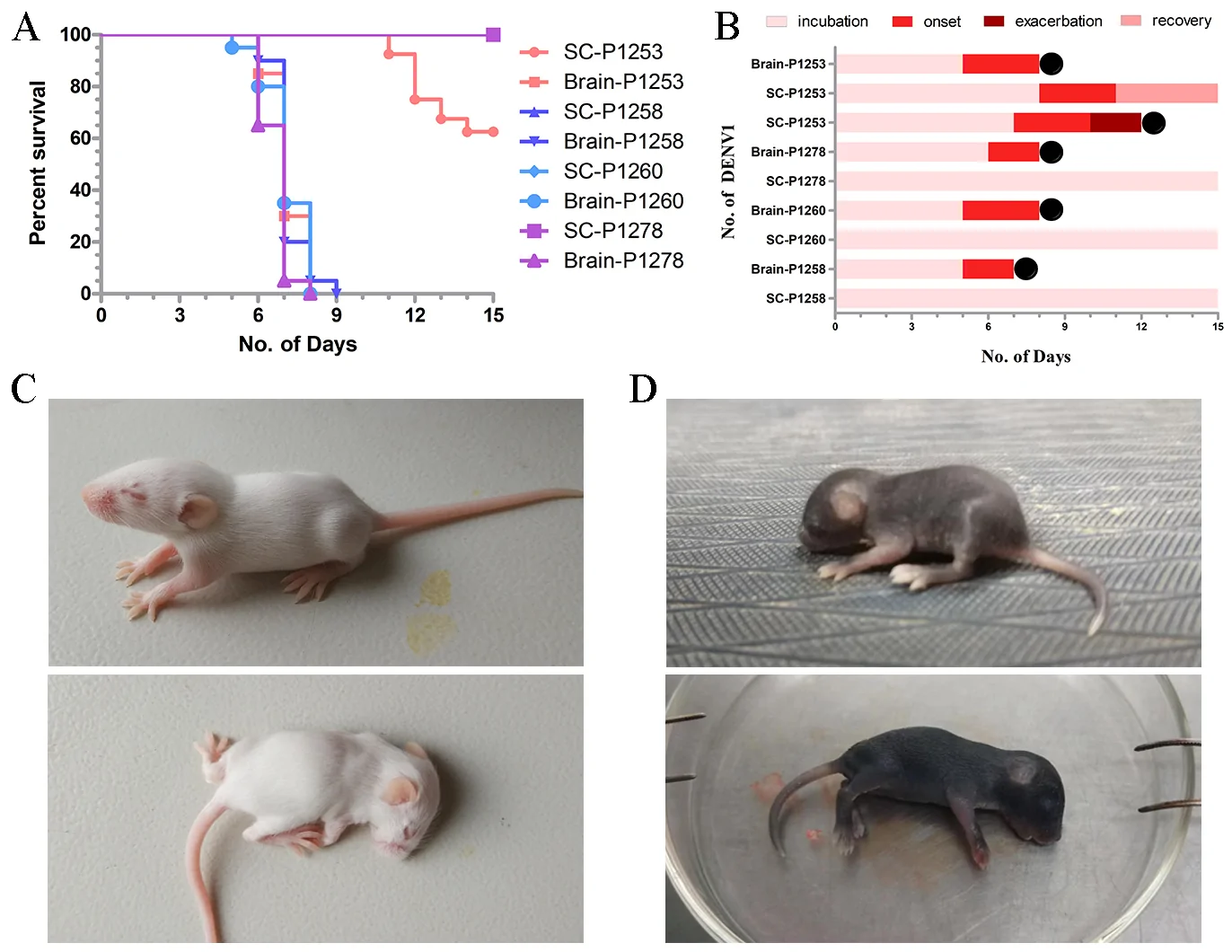
Symptoms and disease course of DENV1 isolates in Kunming and C57BL/6 suckling mice infected by two routes. **(A)** Survival curves of Kunming suckling mice infected with 25 DENV1 strains via intracranial and subcutaneous multi-point injection. Only representative survival curves of P1253 and selected non-neurovirulent strains were displayed. Survival curves represent pooled data from multiple independent replicate experiments. Exact numbers of mice per strain per route are provided in **Supplementary Table 1**. **(B)** Representative disease course of P1253-infected Kunming mice after the two routes; black circles indicate moribund mice. **(A, B)** ”Brain” indicates intracranial injection; “SC” indicates subcutaneous multi-point injection. **(C)** Clinical signs of P1253-infected Kunming mice after subcutaneous injection. **(D)** Clinical signs of P1253-infected C57BL/6 mice after subcutaneous injection. In **(C, D)**, upper panels show mice during the symptomatic phase; lower panels show mice paralyzed and moribund.

A total of 25 DENV1 strains were tested in Kunming suckling mice via intracranial injection (n = 20~26 per strain) or subcutaneous multi-point injection (n = 20~26 per strain), with exact sample sizes for each strain provided in **Supplementary Table 1**. All 25 DENV1 strains caused 100% mortality in Kunming suckling mice after intracranial injection. Only P1253 produced disease (90% morbidity, 35% mortality) via subcutaneous multi-point injection (**Figure 1A**). The two routes differed in incubation period (7–8 days subcutaneous vs. 5–6 days intracranial) and clinical presentation. Intracranial infection produced pronounced arching, shoulder elevation, and hind-limb incoordination, whereas subcutaneous infection produced milder signs: motor retardation, hip drooping, head-tilted resting posture, and tiptoe walking. Thirty-five percent of subcutaneously infected mice progressed rapidly to generalized paralysis and death; the remainder recovered after 2–3 days of illness (**Figures 1B, C**).

C57BL/6 suckling mice infected with P1253 showed highly similar symptoms and disease progression. Intracranial infection caused rapid weight loss, paralysis, and death in all mice. Subcutaneous infection produced an 85% attack rate with more pronounced tiptoe walking and body tremor; 32.5% of mice became paralyzed within 2–3 days, while the rest entered recovery by days 3–5 with residual tiptoe walking (**Figure 1D**).

### 3.2 P1253 crosses the blood-brain barrier and produces histopathological brain damage

**Figure 2:**
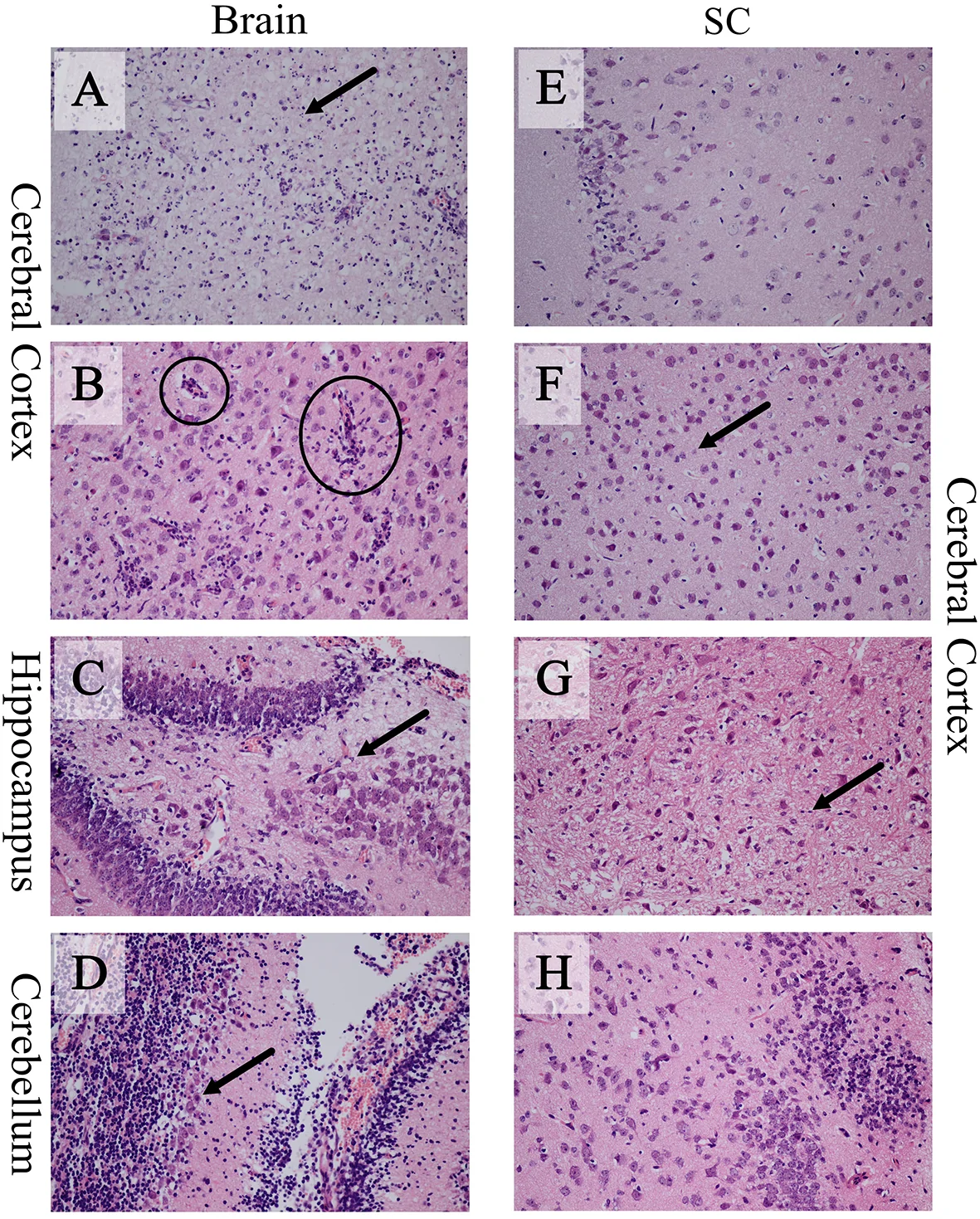
Histopathological brain damage in C57BL/6 suckling mice infected with DENV1 P1253 by two routes. **(A–H)** Hematoxylin and eosin staining, 400×. “Brain” indicates intracranial injection; “SC” indicates subcutaneous multi-point injection. **(A–D)** Intracranial group: cerebral cortex, hippocampus, and cerebellum lesions. **(E–H)** Subcutaneous group: cerebral cortex lesions. **(A, B)** Neuronal apoptosis and necrosis (arrows) and neurotropism (circles). **(C)** Hippocampal microglial pyknosis (arrow). **(D)** Cerebellar molecular layer lymphocytosis and Purkinje cell layer hypercellularity (arrow). **(E)** Disordered cortical neurons. **(F)** Scattered pyknotic neurons (arrows). **(G)** Interstitial edema with focal lymphoid infiltration (arrows). **(H)** Microglial proliferation and focal lymphoid aggregates.

After intracranial infection, P1253 produced lesions in the cerebrum, cerebellum, and hippocampus indistinguishable from other strains: loss of cortical lamination, extensive neuronal apoptosis and necrosis, perivascular lymphocytic cuffing, scattered microglial pyknosis in the hippocampus, and molecular layer lymphocytosis with focal Purkinje cell layer hypercellularity in the cerebellum (**Figures 2A–D**). Only P1253 produced significant brain lesions after subcutaneous infection. These were restricted to the cerebral cortex and were milder, consisting of disordered cortical neuronal arrangement, scattered pyknotic neurons, focal interstitial edema with lymphoid cell infiltration, microglial proliferation, and focal lymphoid aggregates. Perivascular lymphocytic cuffing was absent (**Figures 2E–H**). The cerebellum and hippocampus were unaffected.

### 3.3 P1253 exhibits enhanced neuroinvasion following subcutaneous inoculation

**Figure 3:**
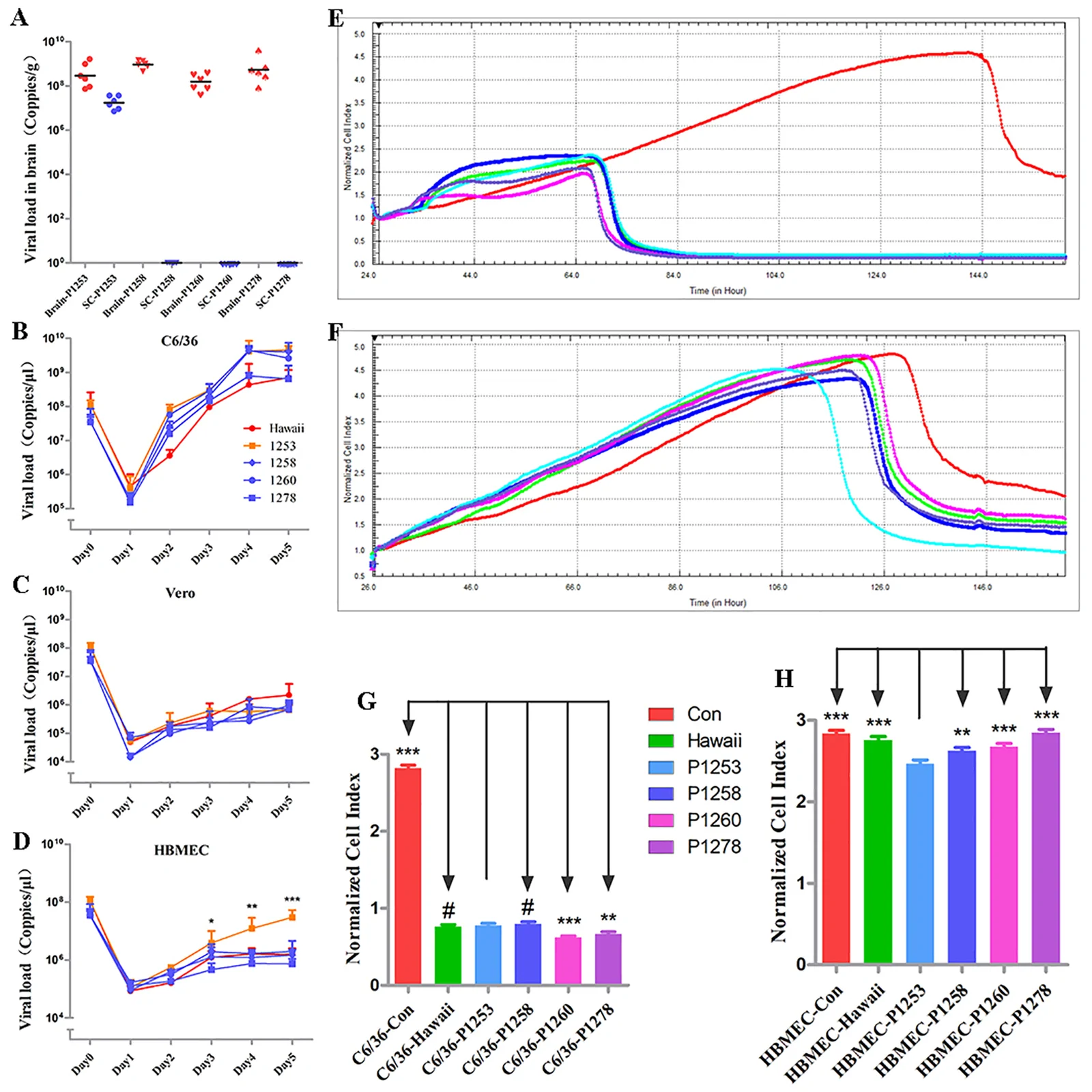
Brain viral load and cellular susceptibility of DENV1 isolates. **(A)** Brain viral loads in Kunming mice infected by two routes with P1253, P1258, P1260, and P1278. “Brain” indicates intracranial injection; “SC” indicates subcutaneous multi-point injection. Brain viral loads were measured in moribund mice or at day 15 post-infection for surviving mice (n=6 per group for viral-load determination). **(B–D)** Viral loads in supernatants of C6/36, Vero, and HBMEC infected with four strains. **(E, F)** Original cell index curves from RTCA of four strains in C6/36 **(E)** and HBMEC **(F)**. **(G, H)** Statistical comparisons against P1253 in C6/36 **(G)** and HBMEC **(H)**. #P > 0.05; **P < 0.01; ***P < 0.001.

To further clarify the neuroinvasive ability of P1253 following peripheral inoculation, we selected P1258, P1260, and P1278 as control strains that produced no disease after subcutaneous injection. Brain viral load was highest in moribund mice after intracranial infection, intermediate in P1253 subcutaneously infected mice, and undetectable in subcutaneously infected P1258, P1260, and P1278 mice (**Figure 3A**). Cell susceptibility analysis of P1253, P1258, P1260, and P1278 showed that P1253 accumulated viral RNA to higher levels in HBMEC than the other strains, with no significant difference in C6/36 or Vero cells (**Figures 3B–D**). RTCA confirmed that P1253 killed HBMEC earlier at equivalent titers (**Figures 3E–H**).

### 3.4 Identification of neurovirulence-associated mutations in P1253

**Figure 4:**
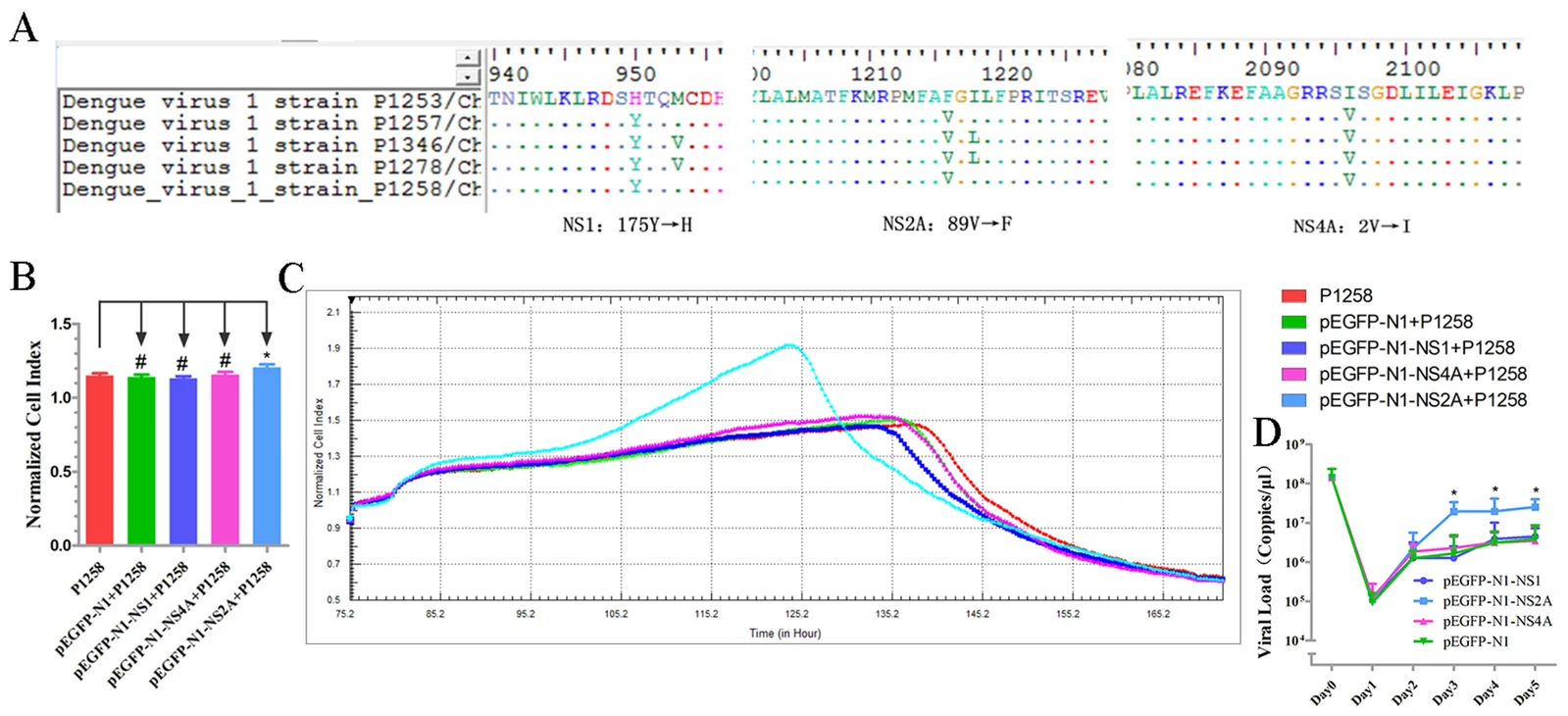
Identification of neurovirulence-associated mutations in DENV1 P1253. **(A)** Alignment of NS1, NS2A, and NS4A coding regions across five strains. **(B–D)** Effects of pre-expression of P1253 mutant proteins (NS1 175Y→H, NS2A 89V→F, NS4A 2V→I) on P1258 cytopathicity **(B, C)** and replication **(D)**. *P < 0.05.

Comparative genomics of P1253 against the four non-neurovirulent strains (P1257, P1258, P1278, P1346) identified three unique mutations: NS1 175Y→H, NS2A 89V→F, and NS4A 2V→I (**Figure 4A**). To isolate the functional impact of individual P1253-specific mutations, we sought P1258 as the reference strain, because P1258 differed from P1253 only at one site in each of NS1, NS2A, and NS4A. Pre-expression of each P1253-derived mutant protein showed that only NS2A 89V→F significantly accelerated HBMEC cell death (**Figures 4B, C**) and enhanced viral RNA accumulation (**Figure 4D**). These results indicate that NS2A 89V→F is associated with enhanced neurovirulence in P1253.

### 3.5 Polymorphism analysis of the DENV1 NS2A 89–96 residue region

**Figure 5:**
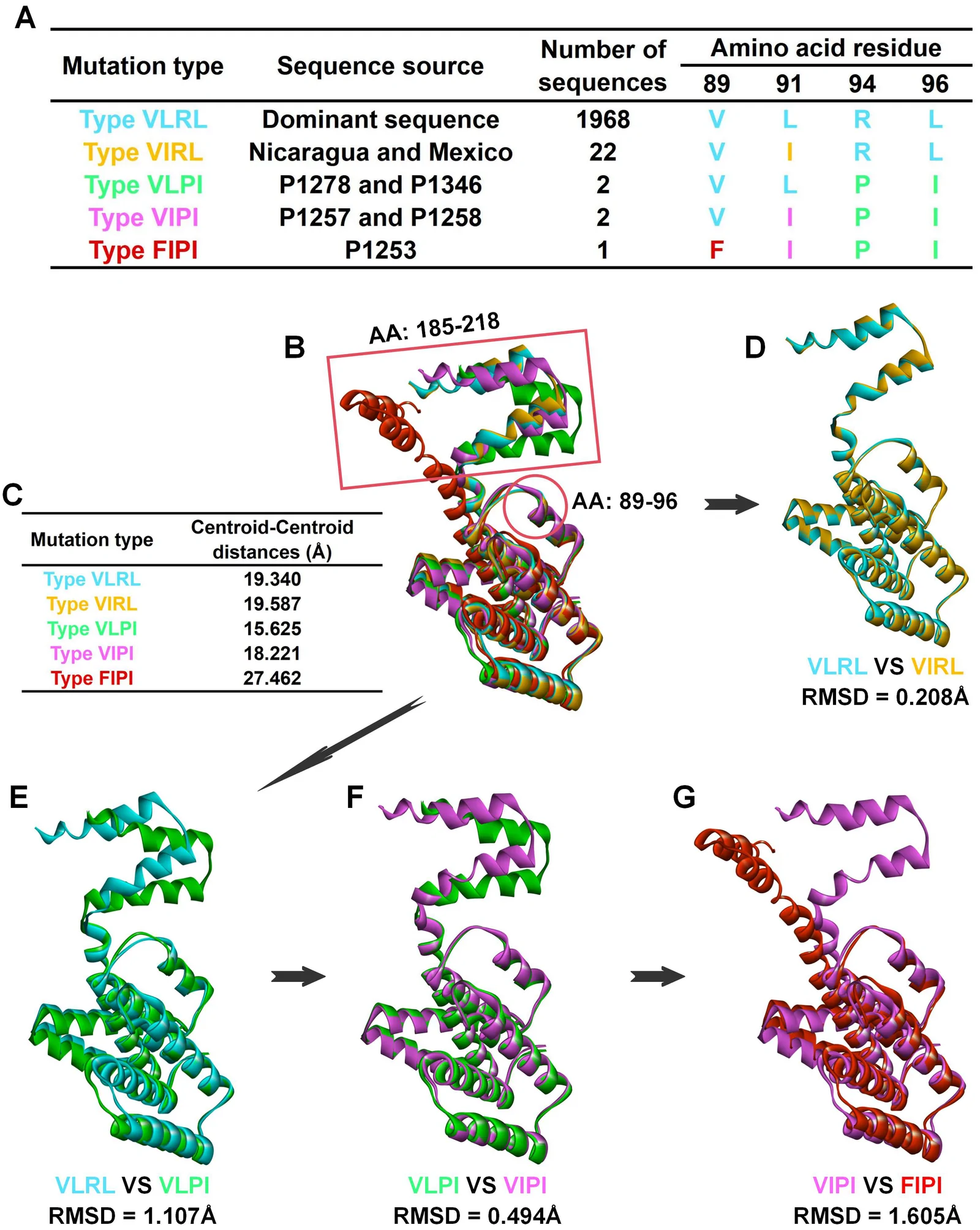
Polymorphism and structural prediction of the DENV1 NS2A 89–96 residue region. **(A)** Distribution of five natural mutant types (VLRL, VIRL, VLPI, VIPI, FIPI). **(B)** Superposition of NS2A structures for the five natural mutant types (VLRL, VIRL, VLPI, VIPI, and FIPI), box indicates residues 185-218, circle indicates 89-96. **(C)** Inter-domain centroid-to-centroid distances between residues 89–96 and 185–218 for each mutant type. **(D)** VLRL versus VIRL superposition. **(E)** VLRL versus VLPI superposition. **(F)** VLPI versus VIPI superposition. **(G)** VIPI versus FIPI superposition.

Analysis of 1,990 complete DENV1 genomes revealed five natural mutant types in the NS2A 89–96 residue region: the dominant VLRL type (1,968 strains, 98.9%), the VIRL type (22 strains, 91I single mutation, circulating in Nicaragua and Mexico during 2008-2016), the VLPI type (P1278/P1346, 94P/96I double mutation), the VIPI type (P1257/P1258, 91I/94P/96I triple mutation), and the FIPI type (P1253, 89F/91I/94P/96I quadruple mutation), demonstrating a progressive accumulation pattern from the baseline to the full quadruple mutant (**Figure 5A**; **Supplementary Figure 1**; **Supplementary Data S2**).

OpenFold3 structural prediction showed that the 89–96 region retained a locally conserved conformation across all five types, whereas the distal 185–218 region underwent significant reorientation (**Figure 5B**). The VLRL-to-VIRL superposition yielded an RMSD of 0.208 Å with nearly identical inter-domain centroid distances (19.340 Å vs. 19.587 Å). Mutation from VLRL to VLPI (RMSD 1.107 Å) and from VLPI to VIPI (RMSD 0.494 Å) produced only mild reorientation (distances 15.625 Å and 18.221 Å, respectively). The VIPI-to-FIPI transition produced the largest RMSD (1.605 Å), with the inter-domain centroid distance increasing markedly to 27.462 Å (**Figures 5C–G**). These computational results suggest that 89V→F is associated with the most pronounced NS2A conformational remodeling among the five natural mutant types.

## 4 Discussion

We screened 25 DENV1 clinical isolates from the 2014 Guangdong outbreak and found that only P1253 caused neurological symptoms and partial mortality in suckling mice after subcutaneous inoculation. This phenotype was reproducible in both Kunming and C57BL/6 strains, indicating robust neurotropism. The subcutaneous route mimics natural hematogenous dissemination, and the ability of P1253 to produce disease via this route suggests efficient peripheral-to-central invasion, unlike intracranial inoculation which bypasses the barrier entirely. Histopathological analysis further showed that P1253 subcutaneous infection produced only focal cortical damage, with the cerebellum and hippocampus spared, whereas intracranial infection caused diffuse encephalitic damage. This cortical-selective injury pattern suggests that either viral load gradients or specific molecular targeting after BBB breach determines the topography of central damage, consistent with existing evidence that DENV can directly infect BBB component cells and induce permeability increases (Calderón-Peláez et al., 2019). However, these results do not directly demonstrate productive infection of CNS parenchymal cells, as the observed neuronal injury may result from indirect effects such as inflammatory mediators or vascular leakage.

In this study, P1253 showed a higher viral RNA levels in human brain microvascular endothelial cells HBMEC. To identify the molecular basis of this cellular tropism difference, we used P1258 as the reference strain in pre-expression experiments and identified the NS2A 89V→F mutation as a candidate determinant associated with enhanced neurotropism. Pre-expression of this mutation significantly enhanced P1258 replication and cytopathic effect in HBMEC. This finding echoes the study by Phu Ly et al., who isolated a DENV-3 genotype III strain from the cerebrospinal fluid of an encephalitis patient that also carried a unique NS2A amino acid substitution and showed enhanced infectivity for human neuroblastoma cells (Phu Ly et al., 2023). Full-genome analyses of DENV-2 and DENV-3 cerebrospinal fluid isolates have also confirmed that the virus can replicate within the CNS (Dhenni et al., 2018; Ngwe Tun et al., 2020b). These cross-serotype observations collectively suggest that NS2A, as a key node among non-structural proteins, is closely related to enhanced DENV neurotropism.

Structural prediction further clarified the potential mechanism by which 89V→F drives enhanced neurotropism. Although progressive mutations in the 89–96 residues had limited impact on local conformation, introduction of 89V→F on the VIPI background (FIPI type) induced the most pronounced distal reorientation of the 185–218 residue region (RMSD 1.605 Å), with the inter-domain centroid distance increasing from 18.221 Å to 27.462 Å, a degree of reorientation far exceeding that of the 91I single mutation or the 94P/96I double mutation. This distal conformational rearrangement is spatially adjacent to the pTMS3 region (residues 69–93, containing the functionally critical R84) described by Xie et al. as regulating viral RNA synthesis and particle assembly (Xie et al., 2013), and also agrees with the study by Wu et al. revealing that NS2A transmembrane segment interactions can reshape virus-induced cytopathic effects (Wu et al., 2017). However, we found that residues 94–96 lie outside the pTMS3 boundary and that the observed 89–96/185–218 relationship represents a computational correlation rather than direct mechanistic proof. These data suggest that 89V→F may remodel NS2A membrane topology or protein-protein interaction interfaces by altering the spatial relationship between pTMS3 and other domains, thereby enhancing replication complex assembly efficiency in HBMEC.

From an evolutionary and epidemiological perspective, NCBI database analysis shows a progressive accumulation from the population baseline VLRL type to the FIPI type in P1253, with the emergence of 89V→F on the 91I/94P/96I background representing the key distinction between P1253 and its contemporaneous counterparts. This mutational accumulation pattern resembles the process reported by Rodriguez-Roche et al., in which the NS1 T164S mutation gradually became an adaptive mutation during the 1997 Cuban epidemic and exacerbated disease severity (Rodriguez-Roche et al., 2005), suggesting that progressive mutation of non-structural proteins may be associated with virulence evolution (Hosen et al., 2026). However, the FIPI genotype is a singleton among 1,990 genomes, and this progressive accumulation pattern, while compatible with stepwise mutational acquisition, does not exclude alternative explanations such as genetic drift, founder effect, or hitchhiking with other selected variants. The HBMEC-specific replication advantage associated with 89V→F is therefore consistent with a putative adaptive role rather than definitive positive selection. Furthermore, the fact that P1253 was isolated from a patient without overt neurological symptoms highlights an important distinction between strain-specific neurovirulent potential and clinical neuroinvasive disease. The suckling mouse model, with its immature immune system and incomplete blood–brain barrier, represents a sensitized system that can unmask latent neurotropic capacities that are effectively suppressed in immunocompetent human adults. Thus, the FIPI genotype may confer a latent neuroinvasive capacity that is not fully manifested in healthy individuals, but could become clinically relevant under conditions of immune compromise, high viral load, or specific host susceptibility. This underscores the importance of routine genomic surveillance of circulating DENV1 strains for NS2A 89–96 polymorphisms, particularly in populations at higher risk for severe or neurological dengue.

This study still has certain limitations. The suckling mouse immune system is immature, and its pathophysiology differs from that of adult human dengue. The neurovirulence observed in this model should be interpreted as indicative of strain-specific neurotropic potential rather than direct evidence of clinical neuroinvasion in humans and further validation in immunocompetent adult animal models is needed. Although pre-expression experiments verified the function of 89V→F, a full-length infectious clone has not yet been constructed by reverse genetics to confirm its causal effect in a single-mutation background. Structural predictions were performed by *de novo* modeling without experimental validation by cryo-EM or X-ray crystallography. Thus, the observed distal reorientation of the 185–218 region represents a computational hypothesis that awaits experimental confirmation. Furthermore, this study tested only HBMEC and did not assess direct infection or replication in CNS parenchymal cells. Future study is needed to determine whether the NS2A 89V→F mutation enhances direct infection of CNS resident cells.

## 5 Conclusion

In summary, this study identified a DENV1 clinical isolate, P1253, capable of inducing neurological disease in suckling mice following subcutaneous inoculation. Integrating animal modeling, cell experiments, comparative genomics, and structural biology analysis, we identified the NS2A 89V→F mutation as a putative driver associated with enhanced neurotropism in this strain. Pre-expression functional assays showed that this mutation promotes neuroinvasion by increasing infection efficiency in BBB-associated cells, while OpenFold3 structural modeling suggested that it drives significant conformational reorientation of the distal NS2A domain on the 91I/94P/96I background. Given the presence of the FIPI genotype among circulating DENV1 strains and its progressive accumulation pattern, close monitoring and molecular characterization of variants carrying this mutation may provide important clues for understanding the association between DENV adaptive evolution and the rising incidence of neurological disease.

## Data Availability

The original contributions presented in the study are included in the article/**Supplementary Material**. Further inquiries can be directed to the corresponding authors.
