# Supplementary material for: NS2A V89F mutation in a DENV1 clinical isolate enhances neurotropism and neuroinvasion
Source: Front Cell Infect Microbiol. 2026 Aug 26;16:1922765. doi: 10.3389/fcimb.2026.1922765 (PMC13553272; doi:10.3389/fcimb.2026.1922765)
Supplement: Supplementary file 1 [file Table3.docx]

Supplementary Material

# 1 Supplementary Data

**Data S1.** OpenFold3 predicted structural coordinates of the five DENV1 NS2A natural mutant types (VLRL, VIRL, VLPI, VIPI, FIPI) in PDB format.

**Data S2.** FASTA file of 1,990 DENV1 NS2A amino acid sequences downloaded from the NCBI Virus database for sequence diversity analysis.

**Data S3.** Sanger sequencing trace files of pEGFP-N1-NS1, pEGFP-N1-NS2A, and pEGFP-N1-NS4A expression constructs for insert and mutation-site verification.

# 2 Supplementary Figures and Tables

**Figure S1.** Multiple sequence alignment of 1,990 DENV1 NS2A amino acid sequences (NCBI Virus database) showing the 89–96 residue polymorphism.

**Table S1.** Clinical background, GenBank accession numbers, sequencing status, and animal experiment outcomes of the 25 DENV1 clinical isolates used in this study.

**Table S2.** OpenFold3 model confidence metrics for predicted structures of all 5 NS2A mutants.

## 2.1 Supplementary Figures

**Supplementary Figure 1.** Multiple sequence alignment of 1,990 DENV1 NS2A amino acid sequences (NCBI Virus database) showing the 89–96 residue polymorphism.
